# Optimizing Ethanol–Water Cosolvent Systems for Green Supercritical Carbon Dioxide Extraction of Muscadine Grape Pomace Polyphenols

**DOI:** 10.1021/acsomega.4c10115

**Published:** 2025-01-29

**Authors:** Arda Tuhanioglu, Sumanjot Kaur, Gabriel Laquete De Barros, Safoura Ahmadzadeh, Renee Threlfall, Ali Ubeyitogullari

**Affiliations:** † Department of Food Science, University of Arkansas, Fayetteville, Arkansas 72704, United States; ‡ Department of Science and Agroindustrial Technology, Faculdade de Agronomia Eliseu Maciel, Universidade Federal de Pelotas, Capão Do Leão, Rio Grande do Sul 96160-000, Brazil; § Department of Biological and Agricultural Engineering, University of Arkansas, Fayetteville, Arkansas 72701, United States

## Abstract

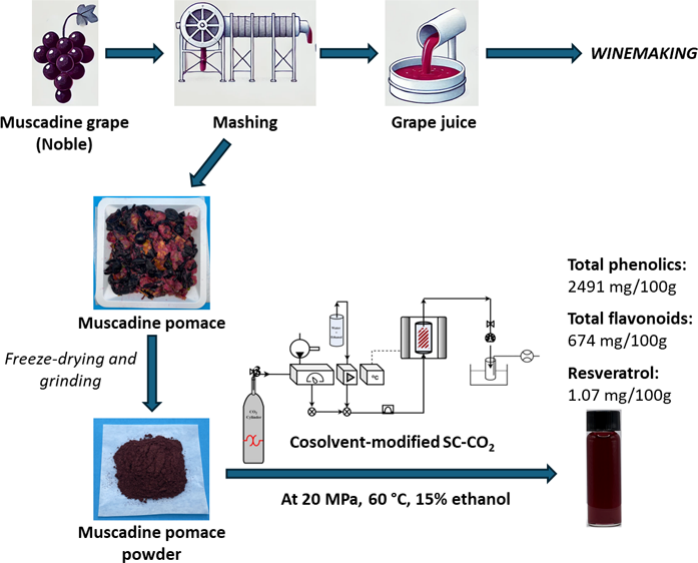

This study evaluated the ethanol–water modified (50%, v/v) supercritical carbon dioxide (SC-CO_2_) for the extraction of polyphenols from muscadine grape (*Vitis rotundifolia* Michgx.) pomace and compared with conventional solvent extractions (ethanol–water and HCl–methanol). The process was optimized with a central composite response surface design consisting of three levels of three independent variables: pressure (20–40 MPa), temperature (40–60 °C), and cosolvent concentration (5–15%) to maximize three responses: total phenolic content (TPC), total flavonoid content (TFC), and resveratrol yields. The optimal conditions were determined as 20 MPa, 60 °C, and 15% cosolvent concentration with TPC, TFC, and resveratrol yields of 2491 mg/100 g, 674 mg/100 g, and 1.07 mg/100 g, respectively. The surface plots indicated that a 15% cosolvent concentration maximized extraction efficiency, producing red-brown colored extracts. In contrast, a 5% cosolvent resulted in poor extractions, yielding yellow-green extracts under all conditions. The yields increased with higher temperatures (i.e., 60 °C) and lower pressures (i.e., 20 MPa). TPC and TFC obtained through cosolvent-modified SC-CO_2_ were similar to those obtained through conventional extractions. Moreover, the resveratrol yield was lower than the HCl–methanol extraction, even though it was not different from any ethanol–water extractions at any solvent-to-solute ratios. The analysis of antioxidants indicated that the ABTS values of the cosolvent-modified SC-CO_2_ extract were lower than those of the HCl–methanol extract. However, there were no significant differences in the DPPH values between the two extracts. Thus, this study optimized the sustainable technology of SC-CO_2_ extraction by employing only food-grade cosolvents—ethanol and water—as a more environmentally friendly method for isolating polyphenols from the underutilized waste product of muscadine grape pomace utilizing statistical methodologies in the extraction process.

## 1 Introduction

According to the Food and Agriculture Organization (FAO) of the United Nations, worldwide grape production reached 73.5 million tonnes in 2021, with the United States being the fourth largest producer with 6.3 million tonnes.^1^ Grapes and wine has been a staple of Mediterranean Europe since the earliest times, both culturally and economically.^2^ Wine grapes accounted for 36.6 million tonnes worldwide in 2022, accounting for 47.4% of the total grape production.^3^ Muscadine grapevines (*Vitis rotundifolia* Michgx.) are a disease-resistant plant native to the U.S. southeastern region that produces various sizes and colors of grapes that are typically think-skinned and seeded.^4,5^ While *V. vinifera* grapevines are grown worldwide for table and wine grape production, they are susceptible to pests and diseases, especially in warm and humid environments where muscadine grapevines can thrive.^4,6^ Muscadine grapes have unique flavors and are grown commercially for fresh and processing (wine, jams, jellies, and juices) markets.^7^ The two primary cultivars for commercial muscadine juice and wine production are Noble (black-skinned) and Carlos (bronze-skinned).^8^

Juice and wine production generates significant waste and byproducts, including grape pomace (skins, flesh/pulp, and seeds) defined as the solid waste remaining after pressing grapes for juice or wine production.^9^ About 20–25% (w/w) of the grape weight becomes grape pomace, leading to over 9 million tons of waste globally every year.^10^ Therefore, juice and wine production facilities have major issues with this waste and are looking for ways to mitigate waste reduction to promote sustainable production practices.^11^ Since muscadine grapes have a very thick and tough skin, muscadine juice and wine production can generate more grape pomace than other *V. vinifera* grapes.^12^ Valuable compounds (i.e., phenolic acids, flavonols, and anthocyanins) can be extracted from muscadine pomace for use in food and nutraceutical products.^13,14^

Polyphenols, organic compounds found abundantly in plant sources, are known for imparting distinct pigmentation and aromas, while also serving as a natural defense system against external threats. Studies have shown that these compounds possess potent antioxidant and anti-inflammatory properties that can help prevent various diseases, including tumor growth inhibition and inducing apoptosis.^15^ Polyphenols represent a vast group of phytochemicals such as phenolic acids, flavonoids, and stilbenes. The polyphenol concentration of muscadine grapes is relatively higher; the content of ellagic acid derivatives was especially reported as unique among *Vitis* varieties.^16^ Anthocyanins, a subclass of flavonoids, are water-soluble pigments found in fruits, flowers, and other parts of plants, providing the characteristics red to blue shown to have positive impacts on cardiovascular and neurodegenerative diseases.^17^ Muscadine skin contains various 3,5-diglucosides anthocyanins such as delphinidin, cyanidin, and petunidin derivatives.^18,19^

Resveratrol is a natural compound that belongs to a subgroup of polyphenols known as stilbenes, which are known for their antioxidant and anti-inflammatory properties. Resveratrol’s anticancer activity was first reported in 1997, which accelerated the studies conducted to explore its potential anti-inflammatory, cardiovascular, and neuroprotective protective effects.^20^ Additionally, resveratrol has a synergistic effect with quercetin and ellagic acid, enhancing its anticancer properties.^21,22^ It has been shown that this particular combination is commonly found in muscadine grape species.^22^ Although resveratrol naturally occurs in numerous plant species, the focus of commercial production is shifting toward biotechnological synthesis from hosts such as *Escherichia coli*, *Lactococcus lactis*, *Streptomyces venezuelae*, *Corynebacterium glutamicum*, and *Saccharomyces cerevisiae*.^23^ Therefore, it is imperative to explore muscadine pomace as a natural and sustainable source of phenolic compounds, incorporating environmentally friendly and effective extraction techniques to recover the high-value compounds at the highest rate.

Incorporating acids (e.g., HCl) or bases (e.g., NaOH) into solvents during extraction is a common practice, which enhances the hydrolysis of bonds that link phenolics to cell wall components, including lignin, cellulose, and hemicellulose.^24,25^ By breaking these bonds, the extraction process releases otherwise inaccessible phenolics, significantly increasing the overall phenolic yield and enhancing the recovery of bioactive compounds. However, acidic and basic solvent extractions have been shown to be more harmful to the environment compared to green alternatives.^26^

Supercritical carbon dioxide extraction (SC-CO_2_) is an eco-friendly method effective in extracting a wide range of compounds from natural sources. It is utilized by food, cosmetic, and pharmaceutical industries to manufacture pure and high-quality extracts with minimal environmental impact.^27,28^ Even though carbon dioxide is a nonpolar solvent, the solvating power of the system can be modified by introducing polar cosolvents (i.e., ethanol and water) to enhance the extraction of phenolics from natural matrices. Our previous studies demonstrated that incorporating a water–ethanol mixture to SC-CO_2_ as a cosolvent substantially increased the extraction of various classes of polyphenols from sorghum bran and rice husk.^29,30^ There were some attempts to extract phenolic compounds from different fruit wastes through SC-CO_2_ approaches.^31^ However, these studies largely emphasize the extraction of specific compounds, such as resveratrol, while neglecting the broader spectrum of phenolics present in grape pomaces.^32,33^ Furthermore, the studies featuring SC-CO_2_ application in muscadine grape products primarily focus on enhancing juice quality and extracting seed oil.^34^ This narrow focus creates a gap in the literature, particularly for muscadine grape pomace, which remains underexplored despite its rich phenolic profile. The studies focusing on the extraction of phenolic compounds from muscadine pomace are scarce. Therefore, the primary objective of this research is to reveal the full potential of muscadine grape pomace by extracting phenolic compounds through the food-grade water–ethanol-modified SC-CO_2_ method and determining their antioxidant capacities. The study also aims to provide a comprehensive understanding of the water–ethanol-modified SC-CO_2_ extraction via statistical tools.

## 2 Materials and Methods

### 2.1 Materials

Muscadine pomace (skin, flesh/pulp, and seeds) was obtained from a research winery. The source of the pomace was from machine-harvest Arkansas-grown, black-skinned muscadine cultivar, Noble. Pectolytic enzymes and heat were applied to the grapes prior to pressing to extract the juice from the pomace. Folin-Ciocalteu’s phenol reagent, sodium carbonate, potassium persulfate, glass wool, glass beads, and Trolox were purchased from Sigma-Aldrich (MO, USA). Gallic acid was purchased from MP Biomedicals (OH, USA), and ethanol was purchased from Fisher Scientific (PA, USA). Resveratrol standard (3,4′,5-trihydroxy-*trans*-stilbene), 2,2-diphenyl-1-picrylhydrazyl (DPPH), and 2,2′-azino-bis(3-ethylbenzothiazoline-6-sulfonic acid) (ABTS) were obtained from TCI (Tokyo, Japan). Carbon dioxide (>99.99%) was supplied by Airgas, Inc. (AR, USA).

### 2.2 Sample Preparation

After removing large residues, such as grape cluster stems, the muscadine pomace was packed and stored at −80 °C. It was then lyophilized for 72 h in a LABCONCO freeze-dryer (MO, USA) at −43 °C and 7.3 Pa. The dried pomace (8.4 ± 0.6% moisture content) was ground to a particle size of ≤500 μm using a KitchenAid Blade Grinder (MI, USA). The final product was stored in airtight containers at 4 °C until further use.

### 2.3 Cosolvent-Modified SC-CO_2_ and Conventional Extractions

SC-CO_2_ extractions were performed using an SC-CO_2_ extractor (Supercritical Fluid Technologies, Inc. from DE, USA) coupled with a cosolvent pump (LL-Class, Supercritical Fluid Technologies Inc., flow rate accuracy of ±2%). A high-pressure, cylindrical stainless-steel vessel with a capacity of 100 mL was filled with the samples. The samples were prepared by mixing 8 g (on a dry basis) of muscadine pomace powder and 80 g of glass beads (3 mm in diameter) to prevent compacting and channeling during the extraction process. The stainless-steel frits sealing the vessel from both ends were fitted with glass wool to prevent clogging. Then, any air in the vessel was removed by flushing with CO_2_ for 5 s. The temperatures of the needle and micrometering valves were set to 80 °C to prevent freezing due to the Joule–Thompson effect. The cosolvent composition was chosen as an ethanol–water (50%, v/v) mixture based on literature data.^30^ The CO_2_ flow rate was set to 3.74 (±2%) g/min (measured at the ambient conditions) after 20 min static extraction. The dynamic extraction time was 3 h in all runs.^32^ The extracts were collected continuously in 40 mL brown glass vials that were kept in an ice bath (∼5 °C). The collected samples were flushed with nitrogen and stored in a freezer at −60 °C until further characterization.

Conventional extractions were carried out at different solvent-to-solute (v/w) ratios using 50% ethanol–water (v/v) to mimic the cosolvent-modified SC-CO_2_ extraction. Muscadine pomace (8 g dry basis) was suspended in the solvents at the solvent-to-solute ratios of 5:1, 10:1, and 15:1 (v/w). The extractions were carried out in a conventional oven at 50 °C for 3 h 20 min. Next, the suspensions were centrifuged at 3220*g* and 4 °C for 10 min. Additionally, acidified methanol extraction was conducted as a conventional laboratory extraction.^27^ Briefly, a 0.1% HCl–methanol solution was prepared and combined with the samples at a 15:1 ratio. The extraction procedure was kept the same as the ethanol–water extraction. The supernatants were analyzed for total phenolic content (TPC), total flavonoid content (TFC), and resveratrol content.

### 2.4 Design of Experiment

The process was optimized via a Central Composite Design (CCD) of Response Surface Methodology (RSM) created in JMP Pro software (version 17.0.0, SAS Software Institute, NC, USA). The design was developed using three independent variables: temperature (°C), pressure (MPa), and cosolvent concentration (%). Each variable was evaluated at three levels: temperature (40, 50, and 60 °C), pressure (20, 30, and 40 MPa), and cosolvent concentration (5, 10, and 15%). The responses measured included total phenolic content (TPC), total flavonoid content (TFC), and resveratrol yield, as shown in Table 1. The design composed a total of 16 runs (2^*k*^ + 2*k* + 2), where *k* denotes the number of independent variables with the center point duplicated. Data belonging to the TFC and resveratrol analyses were normalized by square root and log transformations, respectively. To remediate the lack of fit, Run 5 was omitted only in the resveratrol model.

**Table 1:** Three-Level Central Composite Design for the Ethanol–Water-Modified SC-CO_2_ Extraction of Phenolic Compounds from Muscadine Grape Powder^a^

variable	level		
	–1	0	1
temperature (°C)	40	50	60
pressure (MPa)	20	30	40
cosolvent concentration (%)	5	10	15

a SC-CO_2_: supercritical carbon dioxide. Temperature accuracy: ±0.5 °C.

The quadratic equation was used to fit the data:
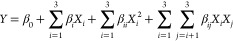
1where *Y* is the response, β_0_ is the constant coefficient, β_*i*_ is the linear coefficient, *X*_*i*_ and *X*_*j*_ represent independent variables, β_*ii*_ is the quadratic coefficient, and β_*ij*_ is the coefficient of interaction.

### 2.5 Total Phenolic Determination

The Folin–Ciocalteu method was employed to determine the TPC of the samples.^35^ Briefly, 100 μL of the sample was mixed with 500 μL of 0.2 N Folin–Ciocalteu’s phenol reagent solution and allowed to react for 5 min at room temperature (23 °C). Subsequently, 400 μL of 0.7 M sodium carbonate solution was added, and the mixture was incubated at room temperature (23 °C) for 2 h. Later, the absorbance of the solution was measured at a wavelength of 760 nm using a spectrophotometer (Milton Roy Spectronic 1201, PA, USA). The measurements were conducted in triplicate, and the results were expressed as milligrams of gallic acid equivalent (GAE) per 100 g of sample with a standard deviation (mg GAE/100 g). The values were obtained using a calibration curve (*R*^2^ = 0.9977) prepared under the same experimental conditions, utilizing gallic acid concentrations ranging from 0 to 200 mg/L.

### 2.6 Total Flavonoid Determination

TFC was determined using an aluminum chloride colorimetric assay.^29^ Briefly, 4 mL of water was mixed with 1 mL of sample, followed by adding 300 μL of 5% sodium nitrite solution. After a 5 min incubation period, 300 μL of 10% aluminum chloride was added and allowed to react for 1 min. Then, 2 mL of 1 M sodium hydroxide solution was added into the mixture. Finally, the total volume was adjusted to 10 mL using distilled water. The absorbance of the resulting solution was measured at 510 nm wavelength using a spectrophotometer (Milton Roy Spectronic 1201, PA, USA). The results were performed in triplicate and expressed as milligrams of catechin equivalent (CE) in 100 g of sample ± standard deviation (mg CE/100 g). The calibration curve (*R*^2^ = 0.9999) was generated with catechin (0–100 mg/L) under the same experimental conditions.

### 2.7 Antioxidant Activities

#### 2.7.1 ABTS Assay

The ABTS assay was conducted to determine antioxidant activity.^35^ For the ABTS assay, a stock solution was prepared by mixing 7 mM ABTS and 2.45 mM potassium persulfate in a 1:2 (v/v) ratio, respectively. The solution was allowed to react for 8 h in the dark at room temperature (23 °C). After incubation, the stock solution was diluted with ethanol to obtain an absorbance of 0.700 ± 0.02 at 734 nm. Subsequently, 100 μL of the extract was mixed with 2 mL of the diluted ABTS solution and incubated for 6 min at room temperature (23 °C). The absorbance of the samples was then recorded at 734 nm and expressed as milligrams of Trolox equivalent (TE) per 100 g of sample ± standard deviation (mg TE/100 g) using a calibration curve (*R*^2^ = 0.9943) prepared using Trolox solutions (10–100 mg/L) under the same conditions.

#### 2.7.2 DPPH Assay

The DPPH assay was also conducted to determine antioxidant activity.^35^ For the DPPH assay, 3 mL of the extract was added to 1 mL of 0.01 mM ethanolic DPPH solution and incubated for 30 min at room temperature (23 °C). Finally, the absorbance was recorded at 517 nm, and the scavenging activity was calculated using the following equation:
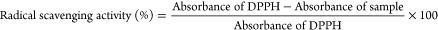
2

### 2.8 Resveratrol Analysis

Resveratrol determination was carried out according to Casas et al.^32,33^ The samples were analyzed using HPLC (Shimadzu Corp., Japan) analysis featuring an SPD-20AV UV/vis detector, SIL—10AF autosampler, and a CTO-20A column oven. The samples were filtered through a syringe filter (0.45 μm) prior to injections. The column (4.6 × 250 mm) utilized in the procedure was a reversed-phase C18 Symmetry column with a particle size of 5 μm (Waters, MA, USA). The mobile phase consisted of water: methanol: acetic acid in a ratio of 75:20:5. A flow rate of 1.5 mL/min was maintained, and 20 μL of the filtered extract was injected. Detection was conducted at a wavelength of 306 nm. Resveratrol was quantified using an authentic standard curve within the concentration range of 7.8–500 mg/L (*R*^2^ = 0.9999) with yields expressed on a dry basis.

### 2.9 Statistical Analysis

Statistical analyses were conducted using JMP Pro (Version 17.0.0, SAS Software Institute, Cary, NC, USA). Tukey’s multiple comparison of means was applied at a significance level of α = 0.05.

## 3 Results and Discussion

### 3.1 Model Fitting and Optimization

Response surface methodology (RSM) enables the comprehension of the effects of independent variables on specific outcomes with minimal experimental runs. This approach facilitates the creation of an empirical model for predicting future operations within the investigated ranges. The results of the RSM design are demonstrated in Table 2, where SC-CO_2_ conditions were optimized for TPC, TFC, and resveratrol yield. As illustrated in Table 3, all responses demonstrated a strong fit with high determination coefficients (*R*^2^) of 0.88 for TPC, 0.98 for TFC, and 0.99 for resveratrol. Furthermore, lack-of-fit tests showed nonsignificance for all responses, providing further evidence of a strong fit (*p* > 0.05).

**Table 2:** Independent Variables (Temperature, Pressure, and Cosolvent Concentration) and Responses (TPC, TFC, and Resveratrol Yield) for the Response Surface Analysis of Muscadine Grape Pomace Powder Extracts Obtained by SC-CO_2_^a^

run	*x*_1_ (pressure MPa)	*x*_2_ (temperature °C)	*x*_3_ (cosolvent concentration %)	TPC (mg/100 g)	TFC (mg/100 g)	resveratrol (mg/100 g)
1	20	50	10	497.93	136.74	0.17
2	30	50	10	405.87	143.84	0.14
3	40	40	15	704.37	145.63	0.17
4	40	60	5	3.69	<0.01	0.01
5	40	50	10	832.83	187.84	0.28
6	40	60	15	1342.06	420.28	0.31
7	20	60	15	2868.83	753.55	0.89
8	20	40	15	1023.61	297.00	0.55
9	30	50	5	9.35	<0.01	0.01
10	30	50	15	1593.57	398.10	0.39
11	30	50	10	595.18	177.86	0.14
12	40	40	5	2.49	<0.01	0.003
13	20	60	5	4.46	<0.01	0.02
14	30	60	10	164.19	93.93	0.23
15	20	40	5	7.88	<0.01	0.01
16	30	40	10	185.12	75.65	0.06

a TPC: total phenolic content, TFC: total flavonoid content, SC-CO_2_: supercritical carbon dioxide.

**Table 3:** Analysis of Variance (ANOVA) and Lack of Fit Tests for the Response Surface Analysis of Muscadine Grape Pomace Powder Extracts Obtained by SC-CO_2_^a^

source	degree of freedom	sum of squares	mean squares	*F*- value	*p*-value	*R*^2^
TPC						
model	6	8020677.3	1,336,780	11.3448	0.0009	0.88
error	9	1060488.5	117,832			
lack-of-fit	8	1042569.5	130,321	7.2728	0.2796	
pure error	1	17919.0	17,919			
TFC						
model	6	1063.8626	177.310	58.5949	<0.0001	0.98
error	9	27.2343	3.026			
lack-of-fit	8	26.3323	3.2915	3.6494	0.3852	
pure error	1	0.9019	0.9019			
resveratrol						
model	4	43.66	10.91	314.31	<0.0001	0.99
error	10	0.34	0.03			
lack-of-fit	9	0.34	0.03	144.18	0.0645	
pure error	1	0.0002				

a TPC:total phenolic content, TFC: total flavonoid content, SC-CO_2_:supercritical carbon dioxide.

Table 4 shows the coefficients of the independent variables for the responses. The main effects were found to be statistically significant for all the responses (*p* < 0.05). In other words, it was observed that pressure, temperature, and cosolvent concentrations exhibited significance across all three responses. Furthermore, the concentration of cosolvent was the most critical variable, with *p*-values approximating zero. Thus, extractions performed at 5% cosolvent concentration resulted in poor TPCs and negligible TFCs and resveratrol yields (Table 2). Furthermore, specific quadratic and interaction effects had significant impacts on different response variables. The quadratic effect of the cosolvent concentration and its interactions with pressure and temperature significantly influenced TPC (*p* < 0.05). On the other hand, the quadratic and interaction effects of the pressure and temperature were nonsignificant (*p* > 0.05). Thus, the quadratic and interaction effects of the pressure and temperature were reduced from the equation (eq 3). For the TFC, the quadratic effect of temperature and the interaction effects of cosolvent concentration were significant (eq 4) (*p* < 0.05). Moreover, the only significant effect, aside from the simple effects, was the quadratic effect of cosolvent concentration on the extraction of resveratrol (eq 5) (*p* < 0.05). It is important to note that the coefficients presented in Table 4 may differ from the values used in the final prediction equations since the equations reflect the optimized model after backward elimination of the nonsignificant effects (*p* > 0.05).
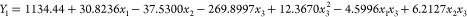
3
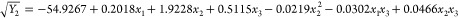
4
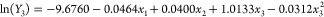
5Where *Y*_1_: TPC, *Y*_2_: TFC, *Y*_3_: resveratrol concentration, *x*_1_: pressure (MPa), *x*_2_: temperature (°C), *x*_3_: cosolvent concentration (%).

**Table 4:** Regression Coefficients for the Response Surface Analysis of Muscadine Grape Pomace Powder Extracts Obtained by SC-CO_2_^a^

	TPC (*Y*_1_)				resveratrol (ln(*Y*_3_))	
variable	coefficient	*p*-value	coefficient	*p*-value	coefficient	*p*-value
constant	–6153.1326	<0.0001	–51.4492	<0.0001	–12.8873	<0.0001
*x*_1_	–18.7846	0.1885	–0.4809	0.1282	0.0321	0.0013
*x*_2_	290.3164	0.0529	1.9778	0.0139	0.1131	0.0018
*x*_3_	–297.8077	0.0003	1.5910	0.0000	1.0642	0.0000
*x*_1_^2^	2.0798	0.3367	0.0132	0.2802	–0.0009	0.5540
*x*_2_^2^	–2.8273	0.2057	–0.0218	0.0984	–0.0005	0.7274
*x*_3_^2^	13.7624	0.1349	–0.0539	0.2719	–0.0279	0.0046
*x*_1_*x*_2_	–1.5036	0.2366	–0.0022	0.7395	–0.0001	0.8590
*x*_1_*x*_3_	–4.5996	0.0910	–0.0302	0.0559	–0.0012	0.4382
*x*_2_*x*_3_	6.2127	0.0348	0.0466	0.0108	–0.0015	0.3335

a *x*_1_: pressure (MPa), *x*_2_: temperature (°C), *x*_3_: cosolvent concentration (%). TPC: total phenolic content, TFC: total flavonoid content, SC-CO_2_: supercritical carbon dioxide.

### 3.2 TPC

**Figure 1:**
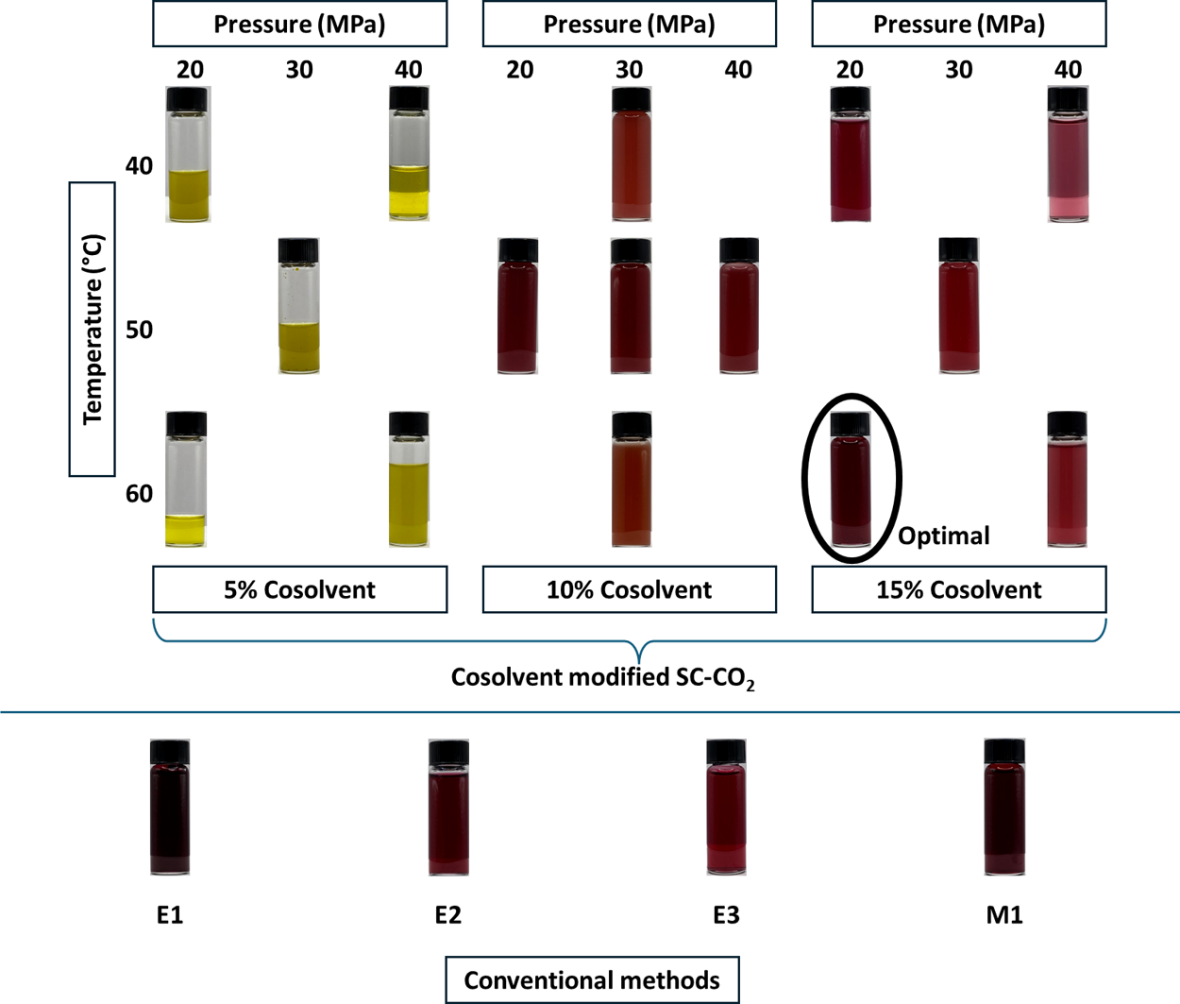
Pictures of the extracts obtained through ethanol–water cosolvent-modified supercritical carbon dioxide (SC-CO_2_) at the designed conditions and conventional extractions performed with different solvents (E1, E2, and E3: 50% ethanol–water (v/v), M1: HCl–methanol) at different solvent-to-solute ratios (E1: 5/1, E2: 10:1, E3: 15/1, and M1: 15/1 v/w).

Table 5 illustrates the yields of the optimal cosolvent modified-SC-CO_2_ and conventional extractions. It has been reported that using aqueous solutions of ethanol, methanol, or acetone produces better results in extracting TPC from red muscadine seed powder compared to using a single-compound solvent system, where ethanol–water ratios between 50 and 70% were ideal.^36^Figure 1 shows muscadine pomace extracts obtained through cosolvent modified-SC-CO_2_ and conventional extractions. Extracts obtained at a 5% cosolvent concentration in modified-SC-CO_2_ were yellow-colored regardless of the pressure or temperature. The lack of color is typically an indication of poor anthocyanin (a subclass of flavonoids) presence, given closer pH values. Coherently, levels of flavonoids measured in the 5% cosolvent runs were negligibly low, as shown in Table 2 (runs 4, 9, 12, 13, and 15). Seabra et al. examined how the cosolvent concentration (ethanol–water) in SC-CO_2_ at 40 °C and 20.9 MPa impacted the extraction of phenolic compounds from elderberry pomace.^31^ In that study, the authors described a “yellow-green” fraction containing low-polarity compounds extracted through the gaseous phase of CO_2_, while the liquid solvent phase was absorbed/adsorbed by the fruit matrix.^31^ Thus, to obtain red-brown colored anthocyanin-rich extracts, cosolvent concentrations higher than 5% were used.

**Table 5:** TPC, TFC, Resveratrol Yield, ABTS, and DPPH Values of the Conventional and Cosolvent-Modified SC-CO_2_ Extractions at Optimal Conditions^a^

solvent	solvent-to-solute ratio (v/w)	TPC (mg/100 g)	TFC (mg/100 g)	resveratrol (mg/100 g)	ABTS (mg/100 g)	DPPH (%)
ethanol–water (50%)	5:1	3294.4 ± 369.10^AB^	1176.3 ± 148.49^AB^	0.99 ± 0.07^B^	339.18 ± 0.74^E^	32.77 ± 2.33^B^
	10:1	4062.90 ± 607.53^AB^	1067.07 ± 454.59^AB^	1.38 ± 0.38^B^	795.87 ± 24.63^C^	79.68 ± 14.67^A^
	15:1	3612.89 ± 10.97^AB^	1254.785 ± 14.78^AB^	1.58 ± 0.28^B^	902.48 ± 27.69^B^	93.19 ± 0.60^A^
HCl–methanol	15:1	4529.29 ± 291.95^A^	1820.35 ± 64.65^A^	3.75 ± 0.04^A^	1204.05 ± 5.03^A^	81.43 ± 0.48^A^
optimum (20 MPa, 60 °C, 15%)	actual	2491.36 ± 533.82^B^	674.30 ± 112.08^B^	1.07 ± 0.25^B^	571.03 ± 31.87^D^	74.58 ± 14.61^A^
	predicted	2444.81 ± 626.05	633.00 ± 121.47	0.84 ± 0.34		

a Means in the same column that are not connected by the same letter are significantly different (*p* < 0.05). TPC: total phenolic content, TFC: total flavonoid content, ABTS: 2,2′-azino-bis-3-ethylbenzothiazoline-6-sulfonic acid, DPPH: 2,2-diphenyl-1-picrylhydrazyl, SC-CO_2_: supercritical carbon dioxide.

**Figure 2:**
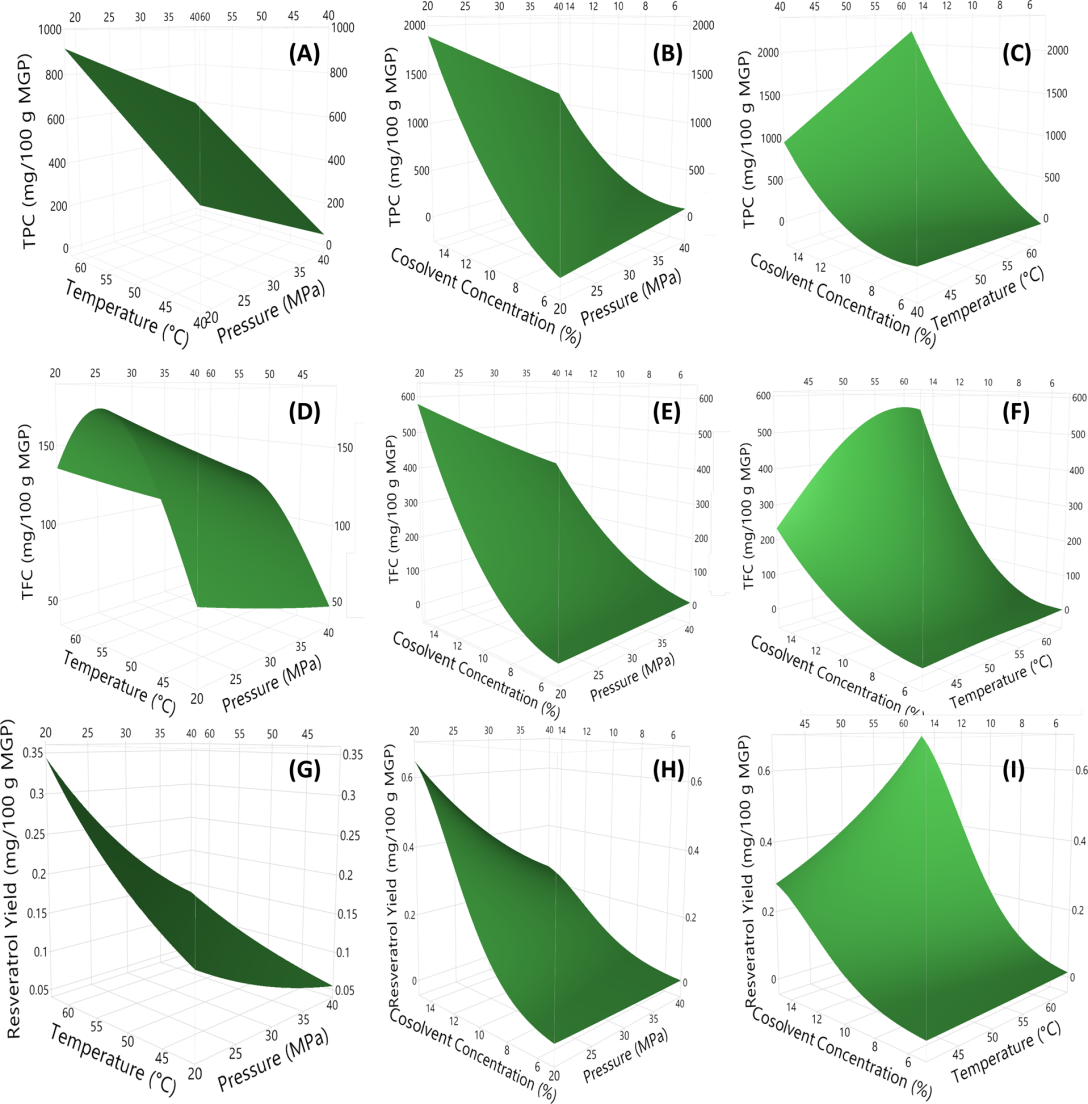
Response surface plots of the TPC: total phenolic content (A–C); TFC: total flavonoid content (D–F); and resveratrol yields (G–I). Graphics A, D, G, were drawn at 10% cosolvent concentration; B, E, H at 50 °C; and C, F, I at 30 MPa). MGP: Muscadine grape pomace (on dry basis).

Figure 2A–C depicts the TPC response surface plots. There was a clear positive correlation between temperature and TPC extraction and a notable negative correlation between pressure and TPC (Figure 2A). At lower cosolvent concentrations (5%), pressure did not have a significant impact on the extraction process. However, at higher cosolvent concentrations (15%), lower pressure was observed to yield superior TPCs (Figure 2B). For instance, at 60 °C and a 15% cosolvent concentration, the application of 20 MPa (Run 7) yielded a higher TPC compared to 40 MPa (Run 6), with values of 2869 and 1342 mg/100 g, respectively. On the other hand, lowering the temperature at low pressure (20 MPa) and high cosolvent concentration (15%) reduced the TPC approximately by one-third (Run 8), which is evident in Figure 2C. Therefore, the optimal conditions for TPC were determined to be 20 MPa, 60 °C and 15% concentration, yielding 2491 ± 534 mg/100 g. Consistently, Oliveira et al. reported the highest global extractions from *V. vinifera* grape pomaces (Merlot and Syrah, separately) at 25 MPa and 60 °C with 15% ethanol as cosolvent concentration.^37^ Several authors recommended the optimal cosolvent concentration of 15% for different grape pomaces, such as Merlot, Syrah, and Cabernet Sauvignon.^37,38^ Furthermore, Oliveira et al. referred to a crossover pattern in the isotherms within the pressure range of 17–19 MPa. It was maintained that the crossover point determines the dominant effect regions, such that, above the crossover pressure, the solute vapor pressure is the governing factor influencing the extraction process.^37^

Table 5 demonstrates conventional phenolic extractions using varying solvents and solvent-to-solute ratios. The type of solvent or the solvent-to-solute ratio did not significantly affect TPC (*p* < 0.05). The TPC obtained via cosolvent-modified SC-CO_2_ at the optimized conditions approximated that of the conventional extractions. Pastrana-Bonilla et al. and Sandhu and Gu reported TPCs of 3073 and 4500 mg/100 g, respectively, for Noble skin, seed, and pulp combined, which are coherent with the current study.^39,40^ Gallic acid and ellagic acid were reported to be the major phenolic acids in the Noble cultivar.^18^ The common consensus is that the majority of the total phenolics are located in the seeds, followed by skin.^39−41^ Sandhu and Gu identified a variety of 88 phenolic compounds in muscadines, comprising 17 in the flesh/pulp, 28 in the skin, and 43 in the seeds.^40^ TPC contents of 396–425 mg/100 g in fresh weights were reported for Noble fruit.^18,39^ Moreover, close TPCs were reported for Noble seed and skin by 3283 and 2549 mg/100 g, respectively.^42,43^

A techno-economic and life cycle assessment of polyphenol extraction from red wine pomace was conducted, comparing solvent extraction with acetone and pressurized liquid extraction using a 50% water–ethanol cosolvent blended with SC-CO_2_ (cosolvent concentration of 75%).^44^ The results revealed that the feasibility and environmental impacts of the studied extraction methods differ based on the solvent-to-solute ratios and the scale of the operation.^44^

### 3.3 TFC

Previously, the major flavonoids, aside from anthocyanins, were reported as catechin, epicatechin, and myricetin for the Noble variety.^18,39^ Based on the surface plots (Figure 2D–F), mild temperatures (50 °C) coupled with low pressures (20 MPa) and high cosolvent concentrations (15%) maximized the TFC. As the concentration of the cosolvent increased, pressure became more significant, similar to the TPC. The surface plots also indicated that elevated temperatures (60 °C) were more advantageous than lower temperatures (40 °C), especially at the high cosolvent concentrations (15%). Thus, the TFC (674.30 ± 112.08 mg/100 g) was maximized at 20 MPa, 60 °C, and 15% within the experimental runs, which was not significantly different than the conventional extractions conducted with ethanol–water (*p* > 0.05) (Table 5). In addition, besides the runs at 5% cosolvent concentration, the lowest TFC was obtained at 30 MPa, 40 °C, with 10% cosolvent resulting in 75.65 mg/100 g (Run 16). In the conventional extractions, higher TFC was measured for HCl–methanol extraction with 1820.35 ± 64.65 mg/100 g on average, though it was not significantly higher than the ethanol–water extractions (*p* > 0.05). The observed phenomenon is likely attributed to the acid’s capacity to break down the bonds between the cell walls and the phenolic compounds, consequently resulting in an augmented yield.^45^ However, it is important to note that despite its common use in analytical practices, acid–methanolic extraction is toxic to humans and constitutes an environmental pollutant.^46^ Another possible explanation is that anthocyanins exhibit greater stability under acidic conditions.^19^ Briefly, anthocyanins are a subgroup of flavonoids with the highest concentrations found in grape skins, while negligible amounts are present in the seeds and pulp. Several studies have indicated that the main anthocyanins in muscadine grapes are the 3,5-diglucosides of delphinidin, cyanidin, and petunidin, making up approximately 90% of the total anthocyanin content.^18,19^

### 3.4 Resveratrol

The skin of the muscadine grape, especially the Noble variety, is known for having a high content of stilbenes (around 7 mg resveratrol equivalent/100 g dry weight) compared to other types of *Vitis* grapes (highest after *V. Vinifera* “Merlot” and *V. ficifolia* “Sangye”).^47^ Most of the resveratrol is found in the skin of the fruit, followed by the seeds.^32,33^ It was reported that the resveratrol content in the dark-skinned variety of muscadine was 5.3 ± 0.7 mg/100 g, while in the bronze-skinned muscadine, it was 3.3 ± 0.4 mg/100 g.^48^ HCl–methanol extraction led to significantly higher resveratrol yield (3.75 ± 0.04 mg/100 g) than conventional ethanol–water (1.58 ± 0.28 mg/100 g) and cosolvent-modified SC-CO_2_ (1.07 ± 0.25 mg/100 g) extractions (*p* < 0.05). No statistical differences were detected between the optimized cosolvent-modified SC-CO_2_ and conventional ethanol–water extractions (*p* > 0.05). The similarity in the surface plots of resveratrol (Figure 2G–I) and TPC (Figure 2A–C) is striking. Similarly, the extraction of resveratrol is more effective when conducted at low pressure (20 MPa) and at a high temperature (60 °C), particularly when using a cosolvent concentration of 15%.

### 3.5 Antioxidant Activities

The antioxidant activities of the cosolvent-modified SC-CO_2_ and conventionally extracted samples were evaluated using both the ABTS and DPPH radical scavenging assays (Table 5). These assays provide insights into the hydrogen atom or electron donation potential of the extracts, reflecting their capacity to neutralize free radicals. All the ABTS values demonstrated significant variability from each other. Specifically, the HCl–methanol extracts displayed the highest ABTS values at 1204.05 ± 5.03 mg/100 g, whereas the conventional ethanol–water extract (at the ratio of 5:1 solvent-to-solute ratio) showed the lowest ABTS values at 339.18 ± 0.74 mg/100 g (*p* < 0.05). On the other hand, the DPPH scavenging activity of the cosolvent-modified SC-CO_2_ (74.58 ± 14.61%) did not differ from the other conventional extractions significantly (*p* < 0.05). The only exception was that the conventional ethanol–water extract at the ratio of 5:1 solvent-to-solute was significantly lower, with 32.77 ± 2.33% (*p* < 0.05). The antioxidant activities of the different fractions (i.e., essential oils vs hydrosols) from Noble skins varied widely.^49^ For instance, the DPPH value of Noble skin essential oils was many folds of its hydrosols.^49^ Thus, the nonsignificant difference in DPPH values could potentially be ascribed to the high nonpolarity of CO_2_ and the comparatively lower polarity of ethanol in contrast to methanol. Consequently, it was anticipated that SC-CO_2_ coupled with ethanol–water would yield superior essential oils extractions, thereby matching the DDPH scavenging activity of the conventional methods.

Numerous studies have shown a lack of correlation between the total phenolic content extracted from plant materials and their antioxidant activity. Pinton et al. developed single and multistage aqueous extraction processes for phenolic extraction from grape pomace (*Vitis vinifera* L. cv. Cabernet Sauvignon).^50^ The results indicated that the developed method outperformed conventional solvent extraction in total phenolic extractions. However, the antioxidant activity demonstrated by the conventionally extracted sample was significantly higher in terms of ABTS.^50^ Similarly, Seabra et al. conducted a comprehensive study examining 30 distinct ternary ratios of SC-CO_2_, ethanol, and water, which aimed to optimize the extraction of total phenolics and to assess the corresponding antioxidant activities from elderberry pomace.^31^ The study detected no direct correlation between TPC and antioxidant activity, although high anthocyanin content was somewhat correlated with higher antioxidant activities, which was attributed to the presence of antioxidant promoters (e.g., rutin) and substances with high antioxidant activity, such as proanthocyanidins. The study concluded that having both ethanol and water were essential for the effective extraction of anthocyanins, although their presence did not have a direct relationship with the antioxidant activity of the extract.^31^ Thus, the detailed composition, environmental conditions during extraction, stability of the extracted phenolics, and their individual contributions and mechanisms to radical scavenging activity must be evaluated for efficient extraction. Although SC-CO_2_ represents a promising eco-friendly alternative to conventional solvent extraction methods, it is imperative to ascertain the optimal levels of antioxidant activity and to identify the primary phenolic compounds involved for targeted applications. This determination is especially pertinent in the context of industrial-scale processing, where a careful evaluation of the trade-off between environmental sustainability and process efficiency must be undertaken.

In conclusion, the cosolvent-modified SC-CO_2_ extraction yielded TPC and TFC comparable to conventional methods, indicating its potential as a viable alternative. Optimal extraction levels were a pressure of 20 MPa, a temperature of 60 °C, and a cosolvent ratio of 15%, yielding 2491 mg TPC and 674 mg TFC per 100 g muscadine pomace. A 15% cosolvent ratio produced effective red-brown extracts, while 5% yielded pale yellow/green extracts. While the resveratrol yield of the cosolvent-modified SC-CO_2_ was lower than that of HCl-methanol extraction (1.07 ± 0.25 vs 3.75 ± 0.04 mg/100 g, respectively), it remained statistically similar to all other conventional ethanol–water extractions. The antioxidant activity analysis revealed lower ABTS values for the SC-CO_2_ extract compared to HCl–methanol, suggesting potential differences in the types of antioxidants extracted. However, the DPPH values were statistically similar between the two methods. Further research is necessary to explore the specific types of polyphenols extracted using the cosolvent-modified SC-CO_2_ method. Additionally, it would be valuable to investigate the bioactivity and potential health benefits of these extracts compared to those conventionally obtained.

This study demonstrated the capability of green SC-CO_2_ technology to replace petroleum-based solvents for polyphenol recovery from the often-overlooked but highly potent phenolic source, muscadine pomace. While the conventional solvent systems were shown to be environmentally hostile, alternative extraction methods and the best solvent/cosolvent combinations for examining polyphenolic sources have been extensively studied; it is essential to consider various economic factors throughout the process. These factors include costs related to procurement, transportation, drying processes, changes in the production chain, and marketing strategies relevant to the specific industry in question. Moreover, ensuring consistency among batches is crucial, as heterogeneity often arises from unclear specifications about cultivars, origin, and storage conditions of fresh muscadine fruits after harvest and pomaces after juice extraction. Such variability results in inconsistent standardization levels, and the distinct local variations may require customized strategies.
